# Diagnostic Accuracy of NS1 ELISA and Lateral Flow Rapid Tests for Dengue Sensitivity, Specificity and Relationship to Viraemia and Antibody Responses

**DOI:** 10.1371/journal.pntd.0000360

**Published:** 2009-01-20

**Authors:** Vu Ty Hang, Nguyen Minh Nguyet, Dinh The Trung, Vianney Tricou, Sutee Yoksan, Nguyen Minh Dung, Tran Van Ngoc, Tran Tinh Hien, Jeremy Farrar, Bridget Wills, Cameron P. Simmons

**Affiliations:** 1 Oxford University Clinical Research Unit, Hospital for Tropical Diseases, Ho Chi Minh City, Viet Nam; 2 Hospital for Tropical Diseases, Ho Chi Minh City, Viet Nam; 3 Center for Vaccine Development, Institute of Science and Technology for Research and Development, Mahidol University, Bangkok, Thailand; 4 Centre for Clinical Vaccinology and Tropical Medicine, Churchill Hospital, University of Oxford, Oxford, United Kingdom; Pediatric Dengue Vaccine Initiative, United States of America

## Abstract

**Background:**

Dengue is a public health problem in many countries. Rapid diagnosis of dengue can assist patient triage and management. Detection of the dengue viral protein, NS1, represents a new approach to dengue diagnosis.

**Methodology/Principal Findings:**

The sensitivity and specificity of the Platelia NS1 ELISA assay and an NS1 lateral flow rapid test (LFRT) were compared against a gold standard reference diagnostic algorithm in 138 Vietnamese children and adults. Overall, the Platelia NS1 ELISA was modestly more sensitive (82%) than the NS1 LFRT (72%) in confirmed dengue cases. Both ELISA and LFRT assays were more sensitive for primary than secondary dengue, and for specimens collected within 3 days of illness onset relative to later time points. The presence of measurable DENV-reactive IgG and to a lesser extent IgM in the test sample was associated with a significantly lower rate of NS1 detection in both assays. NS1 positivity was associated with the underlying viraemia, as NS1-positive samples had a significantly higher viraemia than NS1-negative samples matched for duration of illness. The Platelia and NS1 LFRT were 100% specific, being negative in all febrile patients without evidence of recent dengue, as well as in patients with enteric fever, malaria, Japanese encephalitis and leptospirosis.

**Conclusions/Significance:**

Collectively, these data suggest NS1 assays deserve inclusion in the diagnostic evaluation of dengue patients, but with due consideration for the limitations in patients who present late in their illness or have a concomitant humoral immune response.

## Author Summary

Dengue is a viral infection of humans that is transmitted by mosquitoes. Dengue is a very important public health problem in many developing countries. Recently, new tests to help diagnose patients with dengue have been developed. Evaluating these tests to see how well they perform in different countries and in different health care settings is an important process that helps to guide health care policy on whether these assays are likely to be useful in making a diagnosis, and if so, when best to use them. Our hospital-based results, using two different types of NS1 tests for diagnosing dengue, indicates that these tests are most sensitive when used during the first 3 days of illness and are most likely to be positive if the patient has primary dengue. Our results also show that a positive NS1 test result is a reflection of the amount of virus in the blood, so that patients with high amounts of virus in the blood are more likely to be NS1 positive. Collectively, the results indicate these NS1 tests deserve inclusion in the diagnostic approach to dengue.

## Introduction

Dengue is a major public health problem in many parts of the tropical developing world [1],[2]. Dengue is caused by infection with one of four serotypes of dengue virus (DENV1-4), which are arboviruses belonging to the Flaviviridae family. Although most DENV infections are asymptomatic, a proportion result in clinically apparent disease that varies in severity from mild undifferentiated fever through to more severe syndromes, primarily dengue haemorrhagic fever (DHF) and dengue shock syndrome (DSS). DHF is a vasculopathy characterized by capillary leakage and haematological dysregulation; in severe case hypovolaemic shock (DSS) may develop. There are no licensed vaccines or specific antiviral therapies for dengue, and patient management relies on good supportive care.

Timely, sensitive and specific diagnosis of DENV infection can assist in patient management. Prompt diagnosis of index cases can also facilitate vector control activities in the community so as to mitigate further transmission. NS1 is 55 kDa glycoprotein secreted by DENV infected cells in vitro and in vivo. The function of NS1 in viral replication is not well understood, other than it appears to be essential and might serve to anchor the replication complex to the membrane of the endoplasmic reticulum [3]. NS1 is postulated to contribute to the pathogenesis of dengue. First, in children elevated NS1 plasma concentrations early in illness are associated with more severe disease, possibly reflecting higher viral burdens in these patients [4],[5]. The potential for early NS1 concentrations to predict clinical outcome has been postulated but not assessed [4]. It has been suggested that high NS1 levels may activate complement in solution and/or by directly binding endothelial cells, and may establish foci for immune complex formation leading to complement activation, endothelial damage and capillary leakage [5],[6].

The availability of commercial ELISA assays to detect the DENV NS1 protein in acute plasma provides an additional dengue diagnostic tool to the existing approaches of PCR, serology and, less frequently, virus isolation [7]–[14]. The assessment of NS1 assays as diagnostic tools across a wide range of patient populations and viral serotypes is an important part of the process of identifying where these assays may fit into existing dengue diagnostic algorithms. The purpose of the current study was two-fold. First, to assess the sensitivity and specificity of two commercial NS1 assays, the Platelia ELISA and a lateral flow rapid test (NS1-LFRT), in the context of different viral serotypes, viral burdens and clinical presentations in Vietnamese patients. Second, to assess the specificity of these NS1 assays in patients with other confirmed infections. Our findings suggest that both the Platelia ELISA and NS1-LFRT are specific tools for diagnosing acute dengue, though the sensitivity of both is influenced by the level of viraemia and host humoral immune response.

## Materials and Methods

### Patient enrolment

A series of prospective clinical research studies on dengue are in progress at the Hospital for Tropical Diseases (HTD) in Ho Chi Minh City, Viet Nam. Patients greater than 2 years of age admitted to one of the intensive care units (adult or paediatric) or to one of the general wards with a clinical suspicion of dengue as their primary diagnosis are eligible for enrolment following written informed consent by the patient or guardian. All studies have received approval from the Ethics Committee of the HTD and from the Oxford Tropical Research Ethics Committee. All patients in these studies are assessed daily by a study physician and have serial haematocrit and platelet estimations performed, as well as appropriate sampling for diagnostic serology and virology. Other investigations and clinical management are at the discretion of the attending physicians. After discharge each patient is classified using the current WHO criteria for DF, DHF and DSS. From November 2007 to January 2008, we prospectively tested consecutive acute plasma samples from all children and adults enrolled in these studies.

Plasma samples from patients with another confirmed diagnosis (malaria, enteric fever or leptospirosis) were obtained from stored specimens collected as part of other prospective studies at HTD between 2001 and 2008. The diagnosis of Plasmodium falciparum malaria was made by blood smear. Enteric Fever was diagnosed by blood culture of S. typhi or S paratyphi. Leptospirosis was diagnosed by positive serology (microscopic agglutination test). All samples tested were collected within 10 days of illness onset.

### Dengue diagnostics

A capture IgM and IgG ELISA assay using DENV/JEV antigens and mAb reagents provided by Venture Technologies (Sarawak, Malaysia), was performed as previously described [15]. The interpretation of primary and secondary serological responses was based on the magnitude of IgG ELISA Units in early convalescent plasma samples taking into account the day of illness. The cut-off in IgG ELISA units for distinguishing primary from secondary dengue by day of illness was calibrated using a panel of acute and early convalescent sera from Vietnamese dengue patients that were assayed in the laboratory of Dr Sutee Yoksan using a reference IgM and IgG antigen capture ELISA described previously (the “AFRIMS ELISA”) [16].

DENV loads in plasma were measured using an internally-controlled, serotype-specific, real-time RT-PCR assay that has been described previously [17]. Results were expressed as cDNA equivalents per milliliter of plasma. The dengue IgG indirect ELISA employed uncoated wells and wells coated with recombinant E proteins (Hawaii Biotech, Hawaii) (2 µg/ml). Coated and uncoated wells were blocked with 3% bovine serum albumin for 1 hr. Twofold serial dilutions of plasma, starting at 1/100, were added to each well for 2 hrs, then washed with PBS/Tween20. HRP-conjugated goat anti-human IgG was then added to each well for 2 hrs, after which wells were washed and substrate (TMB) added. The optical density was read at 450 nm. After subtracting the O.D obtained in uncoated wells, the endpoint titre for each plasma sample was defined as the reciprocal of the dilution giving an optical density of 0.3.

The NS1 Platelia and lateral flow rapid tests (NS1-LFRT) were provided by BioRad (Hercules, CA) and were performed according to the manufacturer's instructions. Both NS1 tests were performed in parallel on the same day by the same experienced technician with freshly collected acute plasma samples from each patient. Samples that were defined as equivocal in the NS1 Platelia ELISA assay were repeated. If they were still equivocal they were regarded as being negative. For the NS1-LFRT, each assay strip was independently assessed by the technician conducting the test and a 2^nd^ technician who was blind to the first assessment. During the study, there were no examples of discordance in the interpretation of any NS1-LFRT test. The technicians performing and scoring the NS1-assays were blind to the reference assay results and to any clinical information on the patients.

### The reference algorithm

No single diagnostic assay can diagnose all dengue patients at the various times they may present with symptoms. Consequently, a diagnosis of “confirmed acute dengue” was reached using an algorithm (described in Fig. S1) based on 3 assays; RT-PCR detection of DENV RNA in plasma, and changes in DENV-reactive IgM and IgG levels in paired plasma specimens. In brief, a diagnosis of “lab-confirmed dengue” was made if there was a clinical suspicion of dengue and, a) the RT-PCR assay was positive, b) DENV-IgM seroconversion (i.e. from negative to positive) occurred between paired specimens, c) levels of DENV reactive IgM increased significantly between paired specimens and were very high in the 2^nd^ sample (at least 20% increase in DENV-IgM ELISA Units from 1^st^ to 2^nd^ sample and 2^nd^ sample has at least 20 ELISA Units) , d) there was a four-fold rise in IgG titre to recombinant DENV E proteins measured in indirect ELISA in the presence of significant DENV IgM levels or e) IgG seroconversion was demonstrated in the IgG capture ELISA in the presence of significant DENV IgM levels. The rationale for using two approaches to measuring DENV-reactive IgG is two-fold. The indirect ELISA provides a quantitative measure of IgG titres to recombinant E proteins and is therefore able to detect four-fold changes in IgG levels to the E protein. The IgG antigen-capture assay provides a semi-quantitative measure of IgG levels and is best suited to detecting seroconversion (i.e. from negative to positive). In the context of IgG serology, a diagnosis of “lab confirmed dengue” is only made when the changes in acute/early convalescent IgG levels (i.e. seroconversion or 4-fold change) occur in the presence of detectable DENV-reactive IgM levels. This provides greater specificity for a diagnosis of acute dengue than using IgG measurements alone.

### Statistics

All statistical analysis was performed using Intercooled STATA version 9.2 (StataCorp, TX). Significance was assigned at *P*<0.05 for all parameters and were two-sided unless otherwise indicated. Uncertainty was expressed by 95% confidence intervals. Categorical variables between groups were compared by Fisher's exact test. The *t*-test was used for continuous variables.

## Results

### Characteristics of the study population

The baseline characteristics of the study population of the 138 cases of suspected dengue consecutively enrolled in research studies at the HTD between November 2007 and January 2008 are shown in Table 1. The mean duration of illness prior to the test plasma sample being collected was 3 days (range; 1–6). Patients with a breadth of disease severities and illness durations were represented in the study, including patients with DSS. There were 117 patients diagnosed with “acute dengue”, and 8 cases diagnosed as “recent dengue” according to the laboratory reference algorithm. Of the “recent dengue” cases, 1/8 had DHF and 7/8 had established DSS. Since the clinical picture was so clear in these patients, and was supported by highly suggestive serology, we included these patients in the “acute dengue” category, for a total of 125 acute dengue patients. There were 13 patients in whom there was no evidence of acute or recent dengue.

**Table 1:** Baseline table summarizing key clinical, viral and demographic information on the study population.

Variable	Confirmed dengue (n = 125)	Other febrile illness (n = 13)
	N (%) or Median (range)	N (%) or Median (range)
Male sex	56 (44.8%)	6 (46.2%)
Age (years)	16 (4–42)	12 (7–40)
Day of illness	3 (1–6)	3 (1–6)
**Dengue diagnosis by:**		
PCR alone	11 (9%)	
Serology alone^a^	14 (11%)	
PCR and serology	100 (80.0%)	
**Infecting serotype**		
DENV-1	63 (50.4%)	
DENV-2	20 (16.0%)	
DENV-3	25 (20.0%)	
DENV-4	3 (2.4%)	
unknown	14 (11.2%)	
**Clinical severity**		
DF	49 (39.2%)	
DHFI	16 (12.8%)	
DHFII	40 (32.0%)	
DHFIII	20 (16.0%)	
**Serological status**		
Primary	24 (19%)	
Secondary	93 (74%)	
Indeterminate	8 (6%)	

a includes 8 patients who were diagnosed as “recent dengue” by serology but when considered in the context of their clinical presentations were then defined as confirmed dengue.

### Overall sensitivity of NS1 tests versus reference algorithm

The sensitivity and specificity of each NS1 test was compared against the result of the reference diagnostic algorithm in acute plasma specimens. Samples that were equivocal in the Platelia NS1 assays on first testing (n = 2) were re-tested; one sample was negative whilst the other remained equivocal and thereafter was regarded as negative. Overall, the Platelia NS1 ELISA assay was more sensitive than the NS1-LFRT test for the diagnosis of acute dengue relative to the reference algorithm (Table 2; 83% versus 73%, *P* = 0.047). Against RT-PCR as the reference test, the sensitivity of the Platelia NS1 ELISA assay was greater than the NS1-LFRT (90% versus 79%, *P* = 0.01). There were 4 and 3 patients who were positive in the Platelia NS1 ELISA assay and NS1-LFRT respectively who were negative in the RT-PCR. The specificity of both tests was 100%, albeit the number of patients who had no evidence of acute or recent dengue was small (n = 13) cases.

**Table 2:** Sensitivity and specificity, positive and negative predictive values of each NS1 assay against the gold standard algorithm.

NS1 assays	Study gp (n = )	Acute dengue	NS1 positive	Sensitivity % (95% CI)	Specificity % (95% CI)^a^	PPV % (95% CI)^a^	NPV (%) (95% CI)	*P* value
Platelia NS1	138	125	104	83.2 (75.5–89.3)	100 (86.7–100.0)	100 (97.2–100.0)	38.2 (22.2– 56.4)	0.047
LFRT-NS1	138	125	91	72.8 (64.1–80.3)	100 (91.6–100)	100 (96.7–100)	27.6 (15.6–42.6)	

a one-sided, 95% Confidence Interval.

### Sensitivity of NS1 tests by day of illness

**Figure 1:**
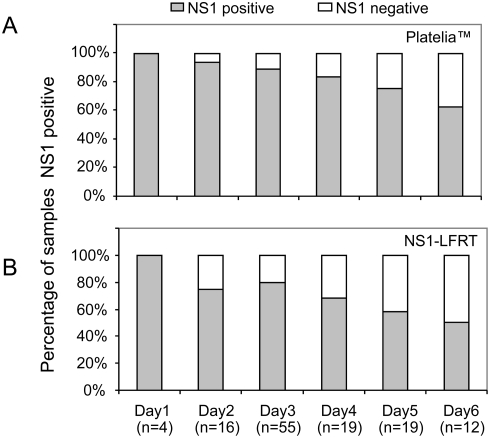
Relationship between day of illness and NS1 sensitivity. Shown is percentage sensitivity of (A) Platelia ELISA and (B) LFRT assays by day of illness in patients with confirmed dengue (n = 125).

The sensitivity of both Platelia and NS1-LFRT tests was influenced by the patient's duration of illness prior to study entry (Fig. 1). Thus, both the Platelia and NS1-LFRT were significantly more sensitive in test samples collected within 3 days of illness onset versus those collected at later times (Table 3).

**Table 3:** Sensitivity of NS1 assays in plasma samples collected within 3 days of illness onset versus those collected at a later time.

Sample group	NS1 status	Platelia % (95% CI)	*P* value^a^	NS1-LFR % (95% CI)	*P* value^a^
Sensitivity of NS1 ≤3 day (n = 75)	NS1 (+)	69	0.002	61	0.01
	NS1 (−)	6		14	
		90.6 (81.7–96.1)		80.0 (69.2–88.3)	
Sensitivity of NS1 >3 day (n = 50)	NS1 (+)	35		30	
	NS1 (−)	15		20	
		70.0 (55.4–82.1)		60.0 (45.1–73.5)	

a Fisher's exact test.

### NS1 sensitivity in primary or secondary infection

In general, NS1 detection was higher in patients with primary dengue than secondary dengue (Table 4). This difference was statistically significant for the NS1-LFRT (*P* = 0.01) and of borderline significance for the Platelia ELISA (*P* = 0.07). The difference in sensitivity between primary and secondary dengue was not associated with the illness day at the time of testing (primary dengue, mean day of illness (SD)), 3.25 (0.21)) versus secondary dengue, 3.66 (0.13), *P* = 0.6)). Reduced sensitivity was also not associated with viraemia levels between primary and secondary dengue cases (log10 mean viraemia (SD), primary: 8.05 (SD:1.40) versus secondary 7.66 (SD:1.53), *P* = 0.2). A possible basis for reduced sensitivity in secondary dengue is that NS1, along with other viral antigens, is sequestered in immune complexes when a substantial level of DENV-reactive IgG is present. To test this hypothesis, we analysed NS1 detection sensitivity in the context of DENV-reactive IgG and IgM antibody in the test sample. The presence of measurable DENV-reactive IgG in the test sample was associated with a significant reduction (*P*<0.001) in NS1 sensitivity in both assays (Table 5). To a lesser extent, DENV-reactive IgM was also associated with a reduction in NS1 sensitivity and was statistically significant in the Platelia assay (*P* = 0.03) (Table 6).

**Table 4:** Sensitivity of NS1 assays in patients with primary and secondary serological profiles.

Sample group	NS1 status	Platelia (95% CI)	*P* value^a^	NS1-LFR (95% CI)	*P* value^a^
Sensitivity of NS1 in primary (n = 24)	NS1 (+)	23	0.07	22	0.01
	NS1 (−)	1		2	
		95.8 (78.9–99.9)		91.7 (73.0–98.9)	
Sensitivity of NS1 in secondary (n = 93)	NS1 (+)	73		61	
	NS1 (−)	20		32	
		78.5 (68.8–86.3)		65.6 (55.0–75.1)	

a Fisher's exact test.

**Table 5:** Sensitivity of each NS1 assay in the presence or absence of measurable DENV-reactive IgG in the test sample.

Status^a^	Total	NS1 Platelia		NS1-LFRT		Sensitivity (% ) (95% CI)	
		Positive	Negative	Positive	Negative	NS1 Platelia	NS1-LFRT
IgG positive	38	23	15	18	20	60.5 (43.4–76.0)	47.4 (31.0–64.2)
IgG negative	87	81	6	73	14	93.1 (85.6–97.4)	83.9 (74.5–90.9)
*P* value						*P*<0.001	*P*<0.001

a IgG in test sample.

**Table 6:** Sensitivity of each NS1 assay in the presence or absence of measurable DENV-reactive IgM in the test sample.

Status^a^	Total	NS1 Platelia		NS1-LFRT		Sensitivity (% ) (95% CI)	
		Positive	Negative	Positive	Negative	NS1 Platelia	NS1-LFRT
IgM positive	35	25	10	23	12	71.4 (53.7–85.3)	65.7 (47.8–80.9)
IgM negative	90	79	11	68	22	87.7 (79.2–93.7)	75.5 (65.4–84.0)
*P* value						*P* = 0.03	*P* = 0.26

a IgM in test sample.

### NS1 sensitivity in relation to viraemia levels

**Figure 2:**
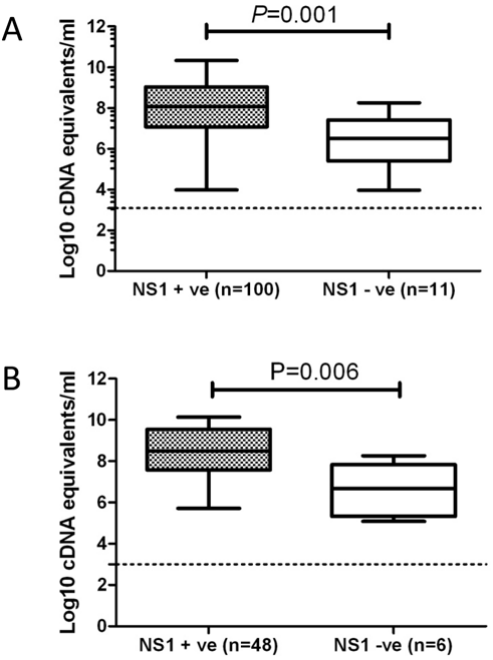
Viral loads by NS1 status in the Platelia ELISA at the time of study enrolment or after 3 days of illness. Shown is the mean (interquartile and range) viraemia level in NS1 positive and NS1 negative (Platelia ELISA) patients with a measurable viraemia (n = 111) at (A) the time of study enrolment or (B) after 3 days of illness durations. The limit of detection of the assay is shown with a dashed line. Viraemia levels were significantly higher in NS1 positive patients relative to NS1 negative patients (Mann-Whitney test). The same observations with regard to viraemia levels were made with the NS1 LFRT (data not shown).

We hypothesised that plasma viremia levels would be associated with the detection of plasma NS1, since NS1, like virions, is a product of infected cells. Accordingly, viremia levels were significantly higher in patients who were NS1-positive at the time of study enrolment versus those who NS1 negative, in both the Platelia assay (Fig. 2A) and NS1-LFRT (data not shown). When viraemia levels were compared in patients with matched illness lengths (3 days), viraemia levels were also significantly higher in NS1-positive patients (Fig. 2B).

### NS1 sensitivity in relation to viral serotype

**Figure 3:**
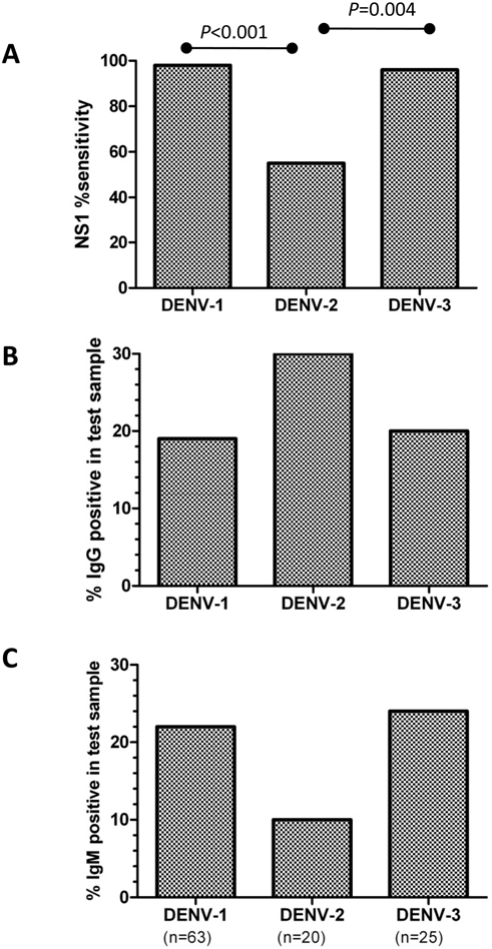
NS1 sensitivity of the Platelia ELISA in the context of viral serotype and humoral immune response. Shown in (A) is the sensitivity of NS1 detection in the enrolment sample according to the infecting serotype identified by real-time RT-PCR (results for DENV-4 not shown as the sample size was small: n = 3). NS1 detection in DENV-2 infected patients was significantly less sensitive than for DENV-1 or DENV-3. The proportion of patients with detectable DENV-reactive (B) IgG or (C) IgM antibodies (measured by capture ELISAs) in the test sample was also related to the infecting serotype. Test samples from DENV-2 infected patients were more likely to have measurable levels of DENV-reactive IgG but not IgM, albeit this was not statistically significant.

The sensitivity of each NS1 assay was considered in the context of the infecting serotype. NS1 detection was significantly reduced in DENV-2 infected patients (55%) relative to DENV-1 (98%; *P*<0.001) or DENV-3 (96%; P = 0.004) infected patients in both Platelia (Fig. 3A) and LFRT (data not shown). The reduced sensitivity of NS1 assays for DENV-2 infected patients could in part be related to the serological response in these individuals; there was a statistically non-significant trend towards more secondary dengue in patients with DENV-2 (85%) than either DENV-1 (76%) or DENV-3 (73%) (*P* = 0.36) and a relatively greater proportion of DENV-2 infected patients had measurable DENV reactive IgG (Fig. 3B), rather than IgM (Fig. 3C), in the test sample. There was also a statistically non-significant trend toward lower viraemia in the test samples from DENV-2 infected patients (DENV-2 log10 mean viraemia (SD); 7.38(1.68)) versus DENV-1 (7.97(1.49)) or DENV-3 (7.79(1.47)). The reduced sensitivity of NS1 detection in DENV-2 infected patients was not due to significant differences in the mean duration of illness at the time of sampling (DENV-1, mean 3.3 days; DENV-2, mean 3.4 days; DENV-3, mean 3.3 days). In summary, NS1 detection was a robust diagnostic test in DENV-1 and DENV-3 infections, but was less sensitive in DENV-2 infections in part because test samples from these patients were generally more likely to have a concomitant DENV-IgG response, suggestive of secondary infection, and lower viraemias, all of which are associated with reduced NS1 detection (see Table 5 and Fig. 2B).

### NS1 specificity in healthy blood donors and patients with other confirmed diagnoses

Since the number of patients with no evidence of acute dengue was small (n = 13) in this study (Table 1), efforts were made to assess the specificity of dengue NS1 assays in patients with other infectious diseases whose transmission geographically overlaps with dengue. To this end, frozen acute (within 10 days of illness onset) plasma samples from patients with culture confirmed enteric fever (S. Typhi, n = 25 and Paratyphi, n = 25), smear positive *P. falciparum* malaria (n = 52), serologically-proven Japanese encephalitis (n = 11) and leptospirosis (n = 12) were tested in parallel by both NS1 Platelia ELISA and NS1-LFRT. In all cases, NS1 tests were negative in these samples.

## Discussion

No single diagnostic assay in isolation is adequately sensitive and specific enough to diagnose all acute cases of dengue. DENV-specific RT-PCR is a robust test during the viraemic febrile phase, but is less sensitive around the time of defervescence, a time when the clinical complications of vascular leakage are most likely to manifest. DENV IgM serology is a simple and robust approach to diagnosis, but this method is not sensitive in the very early stages of disease and strictly, requires paired specimens for definitive laboratory determination. Similarly, IgG serology is not sensitive early in the illness, requires paired specimens and lacks specificity because of cross-reactivity with other flaviviruses. The present study has demonstrated that NS1 detection via ELISA assay or LFRT, particularly in the first 3 days of illness, provides a reasonably sensitive and specific approach to dengue diagnosis in hospitalized patients using a single specimen. Accordingly, we have revised the dengue diagnostic algorithm used by our laboratory to accommodate NS1 testing (Fig. S2).

Studies of the sensitivity and specificity of the Platelia ELISA have been reported previously and, collectively, several prominent themes are evident. First, not every acute dengue case has measurable NS1 antigenaemia and the present study suggests that this is a reflection of the viraemia, with NS1 negative patients having a significantly lower mean viraemia than NS1 positive patients with the same duration of illness history. Second, sensitivity declines with increasing time since the onset of symptoms and this is likely a reflection of decreasing viral burden [7],[10],[13]. Third, Platelia assays are less sensitive in secondary dengue cases [7],[10] and this is consistent with our finding of reduced sensitivity in secondary dengue cases and substantially reduced sensitivity in test samples with a measurable level of anti-DENV IgG. A possible explanation for reduced NS1 sensitivity in the presence of a measurable anti-DENV antibody response is that plasma NS1 is sequestered in immune complexes and that target epitopes are not accessible to either the plate-bound or probe mAb in the NS1 ELISA. Indeed, efforts to dissociate immune complexes can enhance the sensitivity of the Platelia assay [14]. The clinical implications of this in dengue endemic areas are subtle - patients who present to health care facilities more than 3 days after onset of symptoms with clinical signs of vascular leakage, haemorrhage or even DSS may already have an established anamnestic humoral immune response characteristic of a secondary infection and are therefore more likely to be NS1 negative. It is therefore imperative that clinical and laboratory staff understand the limitations of existing NS1 antigen tests and that a NS1 negative assay result does not exclude dengue as a diagnosis. Interestingly, in this study NS1 sensitivity was substantially lower for patients with DENV-2 infections In part, this may relate to the trend for a higher proportion of DENV-2 patients having secondary dengue and having measurable anti-DENV IgG in the test sample. An alternative explanation is that the affinity of the NS1-specific probe and detector mAbs is lower for the lineage (Asian1) of DENV-2 currently circulating in Viet Nam than for DENV-1 or DENV-3; further experiments would be required to address this hypothesis.

An assessment of the NS1-LFRT versus the Platelia ELISA has been conducted previously in adults in French Guiana with a reported overall sensitivity and specificity of 81% and 100% respectively for the NS1-LFRT [7]. Unlike this previous study, we compared the accuracy of each assay, performed in parallel on the same day, in a group of paediatric and adult patients encompassing a broad range of dengue disease severities and considered the results in the context of viral burden and humoral immune response. Overall, the NS1-LFRT assay was modestly less sensitive than the Platelia NS1 ELISA in this study (73% versus 83%) but importantly retained high specificity (100%). The attraction of the NS1-LFRT relative to the Platelia ELISA is its ease of use and speed (15 minutes versus 2 hrs), though this comes at a greater cost per test (∼$10 vs $5). An obvious setting in which to use this assay format is in primary health care clinics for testing of febrile patients presenting early in their illness. In our hospital-based setting, the sensitivity of the NS1-LFRT was 81% in patients admitted within 3 days of illness onset. The ongoing development of specific anti-viral drugs for dengue [18] makes the availability of accurate rapid tests, such as the NS1-LFRT even more important since diagnosing patients quickly and early will provide the greatest window of opportunity for an anti-viral drug to deliver a clinical benefit.

A weakness of the current study is that relatively few of the patients in the prospectively assessed patient population did not have dengue. We compensated for this by including a large number of patients with known alternative diagnoses. The strengths of the current study, and point of difference from published studies, are that we included patients with severe clinical presentations and investigated the relationship between NS1 positivity, viraemia levels, illness history and Ig responses. The finding that viraemia levels are, on average, higher in NS1-positive patients is a novel finding in the context of these commercial assays. The significance of this observation is tied to the widely accepted view that early viraemia levels are associated with disease severity [19],[20]. Thus, NS1 detection may be biased towards detecting those patients who, on average, have the highest viraemias and with relatively higher risks of developing complications during their illness. Future studies should measure the prognostic value of early NS1 measurements for predicting patients at risk of developing severe complications, e.g. DSS.

## Supporting Information

Figure S1 A positive result in any of the first 5 tests is sufficient for a lab diagnosis of confirmed dengue.
(5.58 MB TIF)

Figure S2 Suggested place for NS1 testing in a diagnostic algorithm approach to confirmation of dengue. A positive result in any of the first 6 tests is sufficient for a lab diagnosis of confirmed dengue.
(6.05 MB TIF)

Checklist S1 STARD checklist
(6.59 MB PDF)

Flowchart S1 STARD flowchart for NS1 LFRT
(0.03 MB DOC)

Flowchart S2 STARD flowchart for Platelia
(0.03 MB DOC)
