# Supplementary figures and images for: Diagnostic Accuracy of NS1 ELISA and Lateral Flow Rapid Tests for Dengue Sensitivity, Specificity and Relationship to Viraemia and Antibody Responses
Source: PLoS Negl Trop Dis. 2009 Jan 20;3(1):e360. doi: 10.1371/journal.pntd.0000360 (PMC2614471; doi:10.1371/journal.pntd.0000360)

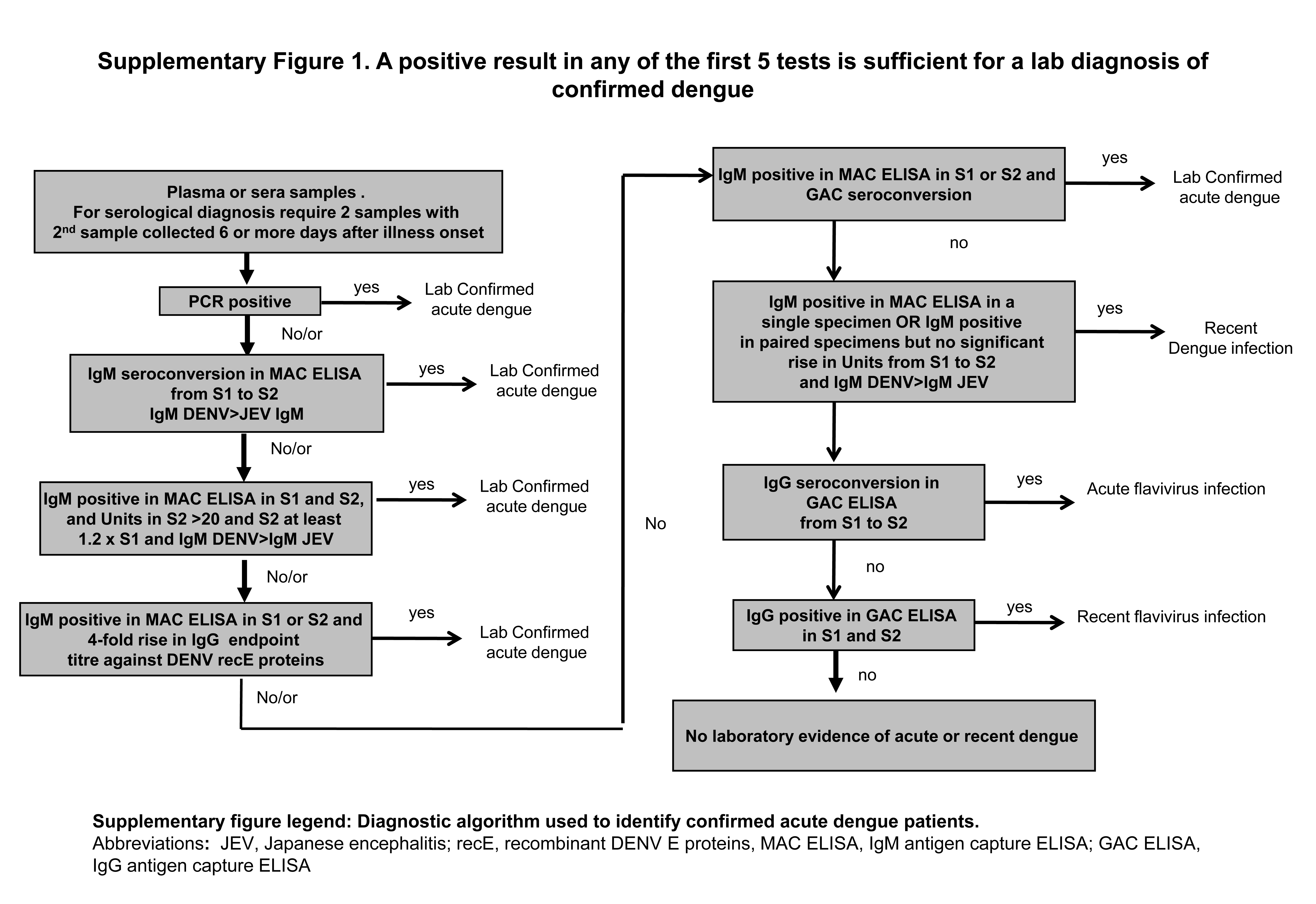

Supplement: Figure S1 — A positive result in any of the first 5 tests is sufficient for a lab diagnosis of confirmed dengue. (5.58 MB TIF) [file pntd.0000360.s001.tif]

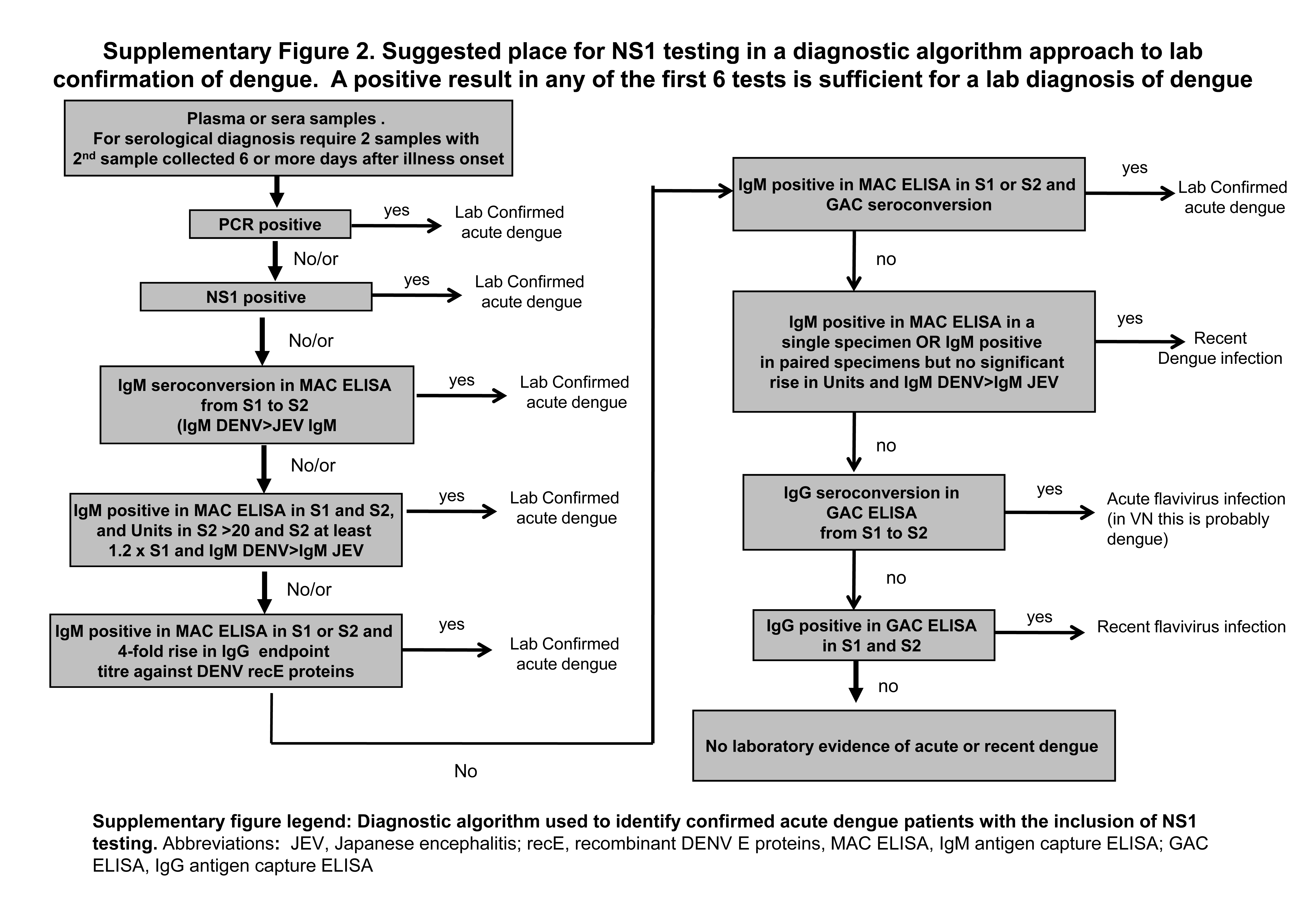

Supplement: Figure S2 — Suggested place for NS1 testing in a diagnostic algorithm approach to confirmation of dengue. A positive result in any of the first 6 tests is sufficient for a lab diagnosis of confirmed dengue. (6.05 MB TIF) [file pntd.0000360.s002.tif]
