# Supplementary material for: Diagnostic Accuracy of NS1 ELISA and Lateral Flow Rapid Tests for Dengue Sensitivity, Specificity and Relationship to Viraemia and Antibody Responses
Source: PLoS Negl Trop Dis. 2009 Jan 20;3(1):e360. doi: 10.1371/journal.pntd.0000360 (PMC2614471; doi:10.1371/journal.pntd.0000360)
Supplement: Flowchart S1 — STARD flowchart for NS1 LFRT (0.03 MB DOC) [file pntd.0000360.s004.doc]

# STARD Flowchart for NS1 LFRT

Eligible Patients (*n* = 138)

Excluded Patients

(*n* = 0)

**NS1 LFRT** (*n=138)*

Positive (*n* = 91)

Negative (*n* = 47)

Equivocal (*n* = 0)

No Reference Standard (*n* = 0)

No Reference Standard (*n* = 0)

**Reference Standard** (*n* = 91)

**Reference Standard** (*n* = 47)

Inconclusive

(*n* = 0)

Inconclusive

(*n* = 0)

Dengue confirmed

(*n* = 91)

Dengue absent

(*n* =0)

Dengue confirmed

(*n* =34)

Dengue absent

(*n* = 13)
